# A case-linkage study of crime victimisation in schizophrenia-spectrum disorders over a period of deinstitutionalisation

**DOI:** 10.1186/1471-244X-13-66

**Published:** 2013-02-20

**Authors:** Tamsin B R Short, Stuart Thomas, Stefan Luebbers, Paul Mullen, James R P Ogloff

**Affiliations:** 1 School of Psychology and Psychiatry, Monash University, 505 Hoddle Street, Clifton Hill Melbourne, 3068, Australia; 2 University of Wollongong, Northfields Avenue, Wollongong NSW, 2522, Australia; 3 Victorian Institute of Forensic Mental Health, Centre for Forensic Behavioural Science, 505 Hoddle Street, Clifton Hill, Melbourne, 3068, Australia

**Keywords:** Psychosis, Victimisation, Violence, Substance use, Deinstitutionalisation

## Abstract

**Background:**

Despite high rates of self-reported crime victimisation, no study to date has compared official victimisation records of people with severe mental illness with a random community sample. Accordingly, this study sought to determine whether persons with schizophrenia-spectrum disorders have higher rates of recorded victimisation than the general population, and to explore whether there have been changes in rates of recorded victimisation over a period of deinstitutionalisation.

**Methods:**

The schizophrenia-spectrum cases were drawn from a state-wide public mental health register, comprising all persons first diagnosed with a schizophrenic illness in five year cohorts between 1975 – 2005. The criminal histories of 4,168 persons diagnosed with schizophrenic-spectrum disorders were compared to those of a randomly selected community sample of 4,641 individuals.

**Results:**

Compared to community controls, patients with schizophrenia-spectrum disorders were significantly more likely to have a record of violent (10.1% *vs.* 6.6%, odds ratio 1.4) and sexually violent victimisation (1.7% *vs.* 0.3%, odds ratio 2.77), but less likely to have an official record of victimisation overall (28.7% *vs.* 39.1%, odds ratio 0.5). Over the approximate period of deinstitutionalisation, the rate of recorded victimisation has more than doubled in schizophrenia-spectrum patients, but stayed relatively constant in the general community.

**Conclusions:**

People with schizophrenic-spectrum disorders are particularly vulnerable to violent crime victimisation; although co-morbid substance misuse and criminality both heighten the chances of victimisation, they cannot fully account for the increased rates. Deinstitutionalisation may have, in part, contributed to an unintended consequence of increasing rates of victimisation amongst the seriously mentally ill.

## Background

People with mental illness typically report that they suffer high levels of crime victimisation [1-3]. Of concern, incidents of victimisation may be particularly detrimental for this population, who are already recognised as vulnerable members of the community [4]. Indeed, crime victimisation can lead to multiple adverse outcomes, and has been associated with increased levels of anxiety and poorer psychosocial functioning amongst people with mental illness [5,6].

Whilst there has been considerably more research on mentally ill persons as perpetrators, rather than victims, of violence [7,8], there is now a growing volume of victimisation research [9]. Indeed, it is increasingly being recognised that people with severe mental illness are in fact more likely to be victims of violence than to commit violent crimes [9,10]. However, one of the most significant limitations with the evidence base in this area is that estimated rates of victimisation amongst mentally ill populations range from 4.3% to 97%, depending upon sample selection, measurement of victimisation, and other methodological variations between studies [11,12]. What the extant literature does suggest is that victimisation is particularly likely to occur amongst mental health clients who are female [5,13], homeless or itinerant [12,14], or those who have more severe psychotic symptoms [2,15], co-morbid substance abuse [15,16], or criminal offending histories [17].

In one of the more comprehensive self-report studies on crime victimisation in the mentally ill, Teplin and colleagues [1] concluded that people with severe mental illness were nearly twelve times more likely than the general population to be victims of violent crime, and were also significantly more likely to be victims of non-violent crimes. Other research has consistently found that rates of crime victimisation are higher amongst the mentally ill than the general population [11].

To date, the vast majority of studies in this area have used self-report measures of victimisation, with only one examining officially recorded rates of crime victimisation [18]. Thus, little is known about the rates of officially recorded victimisation in people with severe mental illness, and how these compare to those of the general population. Of course, self-report measures are recognised as detecting much greater rates of victimisation than official records, as many crimes are not reported to police. Nevertheless, it is crucial to determine the official rates of victimisation amongst the mentally ill, because typically it is only officially recognised ‘victims’ (i.e., those who have their victimisation recorded by the police) who are eligible for victim compensation and support schemes.

In addition to the research on mentally ill crime victims, there has also been considerable public and professional debate about whether rates of crime and victimisation amongst people with severe mental illness have increased following deinstitutionalisation [1,19,20]. While increases in offending by people with mental illness over the period of deinstitutionalisation have been mirrored among the general population [21,22], much less is known about rates and types of victimisation over this period. In fact, there have been no studies to date which have examined rates of victimisation amongst the mentally ill over a period of deinstitutionalisation. There is, however, evidence to show that deinstitutionalisation has led to increased levels of homelessness [23], which is known to be an important risk factor for victimisation [1]. In an era of community-based mental health care, it is important to consider the potential impact of deinstitutionalisation on the risk of crime victimisation in people with severe mental illness.

The current study sought to compare officially recorded rates of crime victimisation amongst schizophrenia-spectrum patients in the Australian state of Victoria with the rates identified in a randomly selected community control sample. The study also compared changes in the rates of victimisation over a thirty year period between a sub-sample of schizophrenia-spectrum cases and matched community controls.

## Methods

This study compared the rates of reported crime victimisation in 4,168 schizophrenia-spectrum patients with 4,641 randomly selected community members, using identical ascertainment methods and measures of victimisation. In keeping with previous Victorian-based research [22,24], this study employed a broad definition of ‘schizophrenia’ to capture not only the various sub-types of schizophrenia, but other chronic primary psychotic disorders (referred to as the ‘schizophrenia-spectrum’ sample). Individual records from the state-wide Victorian psychiatric case register (VPCR) were linked with the Victoria Police Law Enforcement Assistance Program (LEAP) criminal records database.

The VPCR records details of all contacts with the Victorian public mental health system, including community, outpatient, and inpatient services. Diagnoses are made by a psychiatrist at the time of patient discharge (or within one month of admission) and coded according to the *International Classification of Diseases*[25,26]. The LEAP database records all lifetime contacts that an individual has with Victoria Police, including details of all known offences and victimisation incidents.

### Sample selection

The schizophrenia-spectrum sample comprised all persons on the VPCR who were first diagnosed with a schizophrenia-spectrum disorder in the years 1975, 1980, 1985, 1990, 1995, 2000, and 2005. ‘Schizophrenia-spectrum disorder’ was defined as any diagnosis of schizophrenia, schizoaffective disorder, paraphrenia, shared psychotic disorder, delusional disorder, or unspecified non-organic psychosis (ICD-9 codes 295 and 297 plus ICD-10 codes F20, F22, F24, F25 and F29; equivalent DSM-IV-TR codes 295, 297 and 298.9). Organic or transient forms of psychosis, such as substance-induced psychosis or bipolar disorder with psychotic features, were excluded. To account for the inclusion of provisional diagnoses, cases were only included if the initial diagnosis was upheld on at least 75% of subsequent diagnoses, or if there was a clear diagnostic progression culminating in a schizophrenia-spectrum diagnosis. This method of classification has previously been demonstrated to have good diagnostic reliability [27].

The community sample was drawn randomly from the state electoral roll. This database captures approximately 95% of individuals in Victoria and provides an excellent representation of the State’s adult population, since electoral registration is compulsory under state law and the register is updated monthly to remove any deceased persons [28]. Individuals can register on the electoral roll from the age of 17 years, and by law must be registered by the age of 18. Five thousand persons (2,500 males and 2,500 females) were randomly selected from the roll, and full name, gender and age (between 17 and 65 years) were extracted.

### Case-linkage procedures

The initial schizophrenia-spectrum sample comprised 7,177 persons who were first diagnosed with a schizophrenia-spectrum disorder in the cohort years. After excluding those cases which did not meet the above criteria for schizophrenia-spectrum disorder (n=1,263) or which were outside the 17 – 65 year age group (n=1,746), the sample was reduced to 4,168 cases. A full psychiatric history was extracted from the VPCR for each case. Each of the 5,000 cases from the electoral roll was linked with the VPCR databases using a deterministic, then probabilistic, matching procedure (i.e., using exact matches as well as those with different but phonetically matched names). The linkage procedure excluded 286 cases from the original sample (114 due to being outside the 17 – 65 year old age band, 86 due to having only archived psychiatric records, 84 due to irreconcilable multiple name matches in the VPCR, and four due to inaccuracy in date of birth match on the VPCR). Additionally, 71 community cases (1.5%) identified as having a schizophrenia-spectrum diagnosis were removed, making a final community sample of 4,641 cases. Full psychiatric histories were extracted for all eligible cases.

To extract full criminal and victimisation histories, each case in the schizophrenia-spectrum and community samples was linked with the LEAP database using a deterministic match of full name, gender and either date of birth (for those matched on the VPCR) or two-year age band (for those not matched on the VPCR). A subsequent State driver’s licence check was performed to provide further information to assist with the matching procedure (for example, date of driving offence). All schizophrenia-spectrum cases were also searched for on the Victorian Registry of Births, Deaths and Marriages to ascertain dates of death where applicable.

### Matched samples and case–control design

To allow for more detailed temporal analysis of victimisation controlling for cohort effects, a case–control design was employed. Due to restrictions imposed by the Victorian Electoral Commission, only 5,000 community cases were provided and it was not possible to age and gender match each of the schizophrenia-spectrum cases with a community case. Cases first diagnosed with a schizophrenia-spectrum disorder in each of the cohort years (1975, 1980, 1985, 1990, 1995, 2000, and 2005) were matched by age and year of birth with cases in the community sample, whenever a match existed. In cases where multiple matches were found, a matching community case was selected at random. Rates of victimisation in each of the cohort years (percentage of victims) were calculated and compared to the percentages in each cohort of matched community controls.

### Coding of mental health and police records

Mental health records were coded according to type and presence of psychiatric diagnoses (including substance-use disorders, defined as any type of substance dependence, abuse or substance-induced disorders, but excluding nicotine- and caffeine-related disorders).

Police records were coded according to the number and type of victimisation incidents and offences. Victimisation incidents were categorised as ‘violent’ (any offence involving physical contact or harm to the victim, including all assaults and sexual violence) or ‘non-violent’ (all other offences which do not involve physical contact with the victim, including property offences, threats, and non-contact sex offences). Contact sex offences were also considered as a separate sub-category of violent victimisation.

### Ethics and data analysis

The study received ethics approval from Monash University, the Victorian Department of Human Services and Victoria Police, using prescribed methods set out in accordance with current Australian *National Health and Medical Research Centre* guidelines [29]. At no point did the researchers have access to identified data from any of the data sources; contact level data were linked using a unique study identifier.

Descriptive statistics were used to characterise the prevalence of psychiatric diagnoses and police contacts, and to compare the number of victims in each cohort of the matched sample. Binary logistic regression was used to calculate the relative likelihood (odds ratio) of having an official victimisation record in the schizophrenia-spectrum sample compared to the community sample, statistically controlling for potential confounders (age and gender). T-tests were used to compare the mean number of victimisation incidents and mean age at first victimisation between samples. All analyses were conducted using *SPSS Version 19.0*.

## Results

The mean age of the community sample was 39.2 years (*SD*=12.1, 49.4% male), compared to 45.2 years (*SD*=11.2, 63.7%) in the schizophrenia-spectrum sample. On average, the schizophrenia-spectrum group were significantly older (*t*(8869.9) = −24.3, *p*<0.001), and contained significantly more males (χ^2^=181.5, df=1, *p*< 0.001) and significantly more persons with diagnosed substance-use disorders (21.9% *vs.* 1.2%, χ^2^= 909.3, df=1, *p*< 0.001). There were no statistically significant differences in the age of first victimisation between the schizophrenia-spectrum and community comparison groups (31.8 years, *SD*=11.3 *vs.* 31.4 years, *SD*=11.6, *t*(3037)=−1.1, *p*=0.267).

### Prevalence and risk of victimisation in schizophrenia-spectrum disorders

Rates of victimisation are presented in Table 1. One in ten schizophrenia-spectrum patients had a record of violent crime victimisation; this group were significantly more likely than the controls to have a record of violent victimisation, but less likely to have an overall record of victimisation. Rates of sexual violence victimisation were significantly higher in the schizophrenia-spectrum group than the controls (1.7% *vs.* 0.3%, *p*<0.001), with the schizophrenia-spectrum patients being 2.77 times more likely to have a record of sexual victimisation (95% C.I.=1.76 – 4.36). This was particularly marked for females in the schizophrenia-spectrum group.

**Table 1:** Prevalence and odds ratios for official records of crime victimisation comparing the schizophrenia sample with the community comparison sample

	**Schizophrenia group**	**Community group**	OR *	**Adjusted OR (95% CI) **†
	**(N = 4,168)**	**(N = 4,641)**	**(95% CI)**	
Victim of any crime	28.7%	39.1%	0.63	0.50
	(n=1,195)	(n=1,814)	(0.57–0.68)	(0.45–0.56)
			*p *≤ 0.001	*p *≤ 0.001
Victim of a violent crime	10.1%	6.6%	1.58	1.42
	(n=419)	(n=304)	(1.35–1.84)	(1.19–1.70)
			*p *≤ 0.001	*p *≤ 0.001
Victim of a non-violent crime	25%	36.8%	0.57	0.45
	(n=1,040)	(n=1,708)	(0.52–0.63)	(0.41–0.50)
			*p *≤ 0.001	*p *≤ 0.001

* Unadjusted odds ratio.

† Odds ratio adjusted for gender, age and diagnosis of substance-use disorder.

When the number of separate victimisation incidents amassed by victims across the samples were examined, the schizophrenia-spectrum victims had significantly more records of victimisation than the victimised community controls (2.55, *SD*=2.27 *vs.* 1.96, *SD*=1.58, *t*(1946.05) = −7.87, *p*<0.001). Differences were significant for both violent and non-violent offences (mean number of violent incidents in the schizophrenia-spectrum sample = 1.57, SD=0.94, *vs.* 1.36, SD=1.04, *t*(560.48) = −2.58, *p=*0.01; mean number of nonviolent incidents in the schizophrenia-spectrum sample = 2.15, SD=1.77, *vs.* 1.82, SD=1.33, *t*(1750.38) = −5.23, *p*≤0.001).

### Effects of co-morbid substance-use disorders

The schizophrenia-spectrum sample was further divided into those patients with (*n*=913) and without (*n*=3,255) a diagnosed co-morbid substance-use disorder. The relative prevalence of victimisation records in the co-morbid, schizophrenia-spectrum-only and community samples are presented in Table 2. Over one third of all patients with co-morbid substance-use disorders had an official record of crime victimisation, and one in five (20.4%) had a record of violent victimisation. In comparison, 1.2% (n=55) of the community sample had been diagnosed with a substance-use disorder, and there was no significant difference between the proportion of victimisation incidents in those community members with (n=27) and without (n=28) substance-use disorders (χ^2^= 2.34, df=1, *p*=0.126)^1^[1]. Table 2 demonstrates that both the schizophrenia-spectrum-only and co-morbid groups were significantly more likely than the controls to have a record of violent victimisation, controlling for the potentially confounding effects of age and gender.

**Table 2:** Comparison of violent crime official victimisation records in the schizophrenia-only, co-morbid, and community samples

	**Record of any crime victimisation**	**Record of violent crime victimisation**	**Odds ratios for violent victimisation (compared to community sample)**
Schizophrenia-only sample N = 3,255	24.7%	7.2%	OR* = 1.39
	(n=805)	(n=233)	95% C.I. = 1.15 – 1.68
Co-morbid sample N = 913	42.7%	20.4%	OR* = 3.79
	(n=390)	(n=186)	95% C.I. = 3.07 – 4.68
Community control sample N = 4,641	39.1%	6.6%	–
	(n=1814)	(n=304)	

*Odds ratio adjusted for gender and age. Data presented are based on official police records.

When age and gender were controlled for, co-morbid schizophrenia-spectrum patients were 1.91 times more likely to have a record of any victimisation than schizophrenia-spectrum patients without a known substance-use disorder (95% C.I.=1.63 – 2.25), and 2.59 times more likely to have a record of violent victimisation (95% C.I.=2.08 – 3.24). Compared to the community control group, the co-morbid schizophrenia-spectrum group were 6.42 times more likely to have a record of sexual violence victimisation (95% C.I.=2.92 – 14.12).

### Relationship between offending and victimisation

Schizophrenia-spectrum patients who had been charged with a criminal offence (*n*=807) were 4.80 times more likely (95% C.I.=3.71 – 6.20, *p*≤0.001) than schizophrenia-spectrum non-offenders (n=2,413) to have a record of violent victimisation, and 3.07 times more likely (95% C.I.=2.55 – 3.69, *p*≤0.001) to have a record of non-violent victimisation, after controlling for the effects of age, gender and substance-use disorders. There was also a significant association between offending and victimisation in the community sample. Controlling for the effects of age, gender and substance-use disorders, community members who had been charged with a criminal offence were 2.63 times more likely than those without an offence history to have a record of non-violent victimisation (95% C.I.=2.11 – 3.28), and 5.2 times more likely to have a record of violent victimisation (95% C.I.=3.90 – 6.93). One caveat here, however, is that many people first come to the attention of public mental health services due to their behaviour and/or actions attracting the attention of police who then have them assessed by, or divert them into, health services. As such, because we considered lifetime histories of criminal victimisation, it is possible that a small proportion of the officially recorded victimisation experiences occurred prior to a schizophrenia spectrum diagnosis having been officially made/recorded.

### Victimisation over a period of deinstitutionalisation

**Figure 1:**
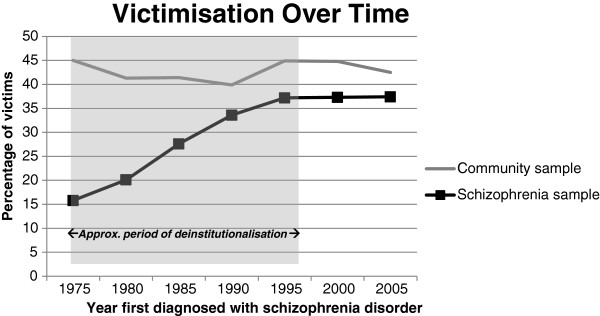
**Victimisation rates over a period of deinstitutionalisation in the matched case–control design for schizophrenia patients first diagnosed between 1975–2005. **See ‘Limitations of the Study’ in the Discussion section.

Figure 1 shows the rates of victimisation in the schizophrenia-spectrum and community samples over time, from patients first diagnosed in 1975 to those first diagnosed in 2005. Whilst rates have remained relatively constant over time in the community sample (between 40 – 45%), there has been a steady increase in lifetime victimisation records amongst those in the schizophrenia-spectrum sample, from 15.3% in those first diagnosed in 1975 up to 37.4% in those first diagnosed in 2005. These rates appear to have reached a plateau at around 37% since 1995.

## Discussion

This study compared officially recorded rates of crime victimisation between a large sample of persons with schizophrenia-spectrum disorders and a randomly selected community sample that had never been diagnosed with a schizophrenic illness. Persons with schizophrenia-spectrum disorders were significantly less likely to have an official record of non-violent victimisation, but more likely to have a record of violent crime victimisation. Furthermore, when victimised, they were more likely than the community sample to experience repeated instances of victimisation. Although co-morbid substance-use disorders were associated with more frequent victimisation records, even schizophrenia-spectrum patients without a substance-use disorder were more likely than controls to have a record of violent victimisation.

This was also the first study to compare rates of crime victimisation over a period of deinstitutionalisation. Alarmingly, the rates of victimisation amongst schizophrenia-spectrum patients were more than double in those first diagnosed in 1995 compared to those first diagnosed in 1975, whilst rates in the community sample remained constant. These findings provide further indication that people with severe mental illness are vulnerable to harm and victimisation in the community, and bring into question whether the current community-based mental health care model is able to manage and protect this vulnerable population.

### Limitations of the study

An inevitable degree of error is incurred when using case-linkage procedures. However, the chance of these results being impacted by such error is minimised by the large sample size (N=8,809) and exclusion of cases where there was some indication of linkage inaccuracy. An additional limitation of case-linkage is that data interpretation is restricted by the accuracy and completeness of data available. Information contained in the databases was not collected for research purposes and is limited in scope, precluding the consideration of certain variables (such as socioeconomic status) in this study. It is also possible that relatively minor instances of crime victimisation which occur in institutional settings (such as prisons) may be dealt with internally and not reported to police, therefore excluding these incidents from the source database. However, if this is the case, then rates of victimisation would be expected to be even greater in the schizophrenia-spectrum sample, as this group are more likely to be institutionalised.

It is well-recognised that official records are an under-estimate of the true crime rate [30], and the rates reported here will under-estimate the true levels of victimisation in the community. However, the aim of this study was not to identify absolute rates of community victimisation, but rather to ascertain the level of officially recorded victimisation amongst schizophrenia-spectrum patients, and compare this with that recorded by the general population (using identical methods). Although there is currently no research comparing crime reporting by people with and without mental illness, there is reason to suspect that people with severe mental illness may be even less likely than others in the community to report victimisation experiences to the police [30]. As such, any significant findings of more frequent victimisation in the schizophrenia-spectrum sample can be interpreted with confidence, as they are likely to only underestimate the true difference between these groups.

In addition to crime victimisation, the rate of substance-use disorders in both groups is likely to be underestimated here, due to less than perfect detection and recording of substance problems on the VPCR. It may further be the case that substance-use disorders are even less likely to be identified in the community sample, as this group were typically not in regular contact with the public mental health system and arguably had less opportunity for diagnosis and detection of substance problems. However, it is well-recognised that people with major mental illness engage in more prevalent substance misuse than the general community, and this general pattern was reflected in the current study [31,32].

Finally, it must be noted that the current study was conducted in a country with relatively low rates of crime, which now operates under a community-based mental health system. These results therefore may not be directly generalisable to other jurisdictions with different base rates of crime, or significant variations in policing and mental health systems. We note also that the base rate of violent victimisation is low, and that the practical significance of statistically significant differences in odds ratios must be considered in this context. Nevertheless, given the serious consequences of violence victimisation, even relatively small numerical differences may have a powerful affect on individuals and communities.

### Crime victimisation in people with schizophrenia-spectrum disorders

In contrast with previous research [18], the current study found that people with schizophrenia-spectrum disorders are less likely than the general community to have an official record of victimisation, and also have a lower rate of recorded non-violent victimisation. This finding appears to be at odds with the general hypothesis that people with mental illness are more vulnerable to both violent and non-violent forms of crime [1,15]. One possible explanation for these results is that people with schizophrenia-spectrum disorders are less likely to report non-violent or less serious victimisation to police than other persons in the community. Contemporary research also shows that offenders or persons who have had prior negative experiences with police are less likely to report their own victimisation experiences [30], a finding which may be particularly pertinent for schizophrenia-spectrum patients given the increased rates of offending and police contact found among this population [7-9]. Further, it is plausible that certain psychotic symptoms (such as paranoia or persecutory delusions) will further discourage schizophrenia-spectrum patients from reporting victimisation experiences to authority figures. It is also recognised that many people are motivated to report non-violent property crimes by requirements of their insurance policies [33]; if persons with severe mental illness are less likely to have comprehensive insurance (or cannot afford to pay excess costs), it follows that they would be less likely to report such crimes. Thus, it is perhaps reasonable that recorded rates of victimisation overall are lower in people with schizophrenia-spectrum illnesses. Further research could consider a more nuanced comparison of different types of non-violent crime to tease out where the differences lie and therefore to provide a focus for enhanced criminal justice supports and interventions.

On the other hand, the recorded rates of violent victimisation were significantly higher amongst the mentally ill group. Overall, one in ten people with a schizophrenia-spectrum disorder had been recorded as a victim of violent crime, and this group were more likely to have a record of victimisation than the community controls. Of concern, the risk of victimisation seems to be particularly high for sexual offences; patients with schizophrenia-spectrum disorders were nearly three times more likely to have a record of sexual violence victimisation. Moreover, victims with schizophrenia-spectrum disorders had more frequent and repeated victimisation incidents than community controls.

The lifetime prevalence of recorded violent victimisation for persons with schizophrenia-spectrum disorders was 10%, a rate markedly lower than that reported in most self-report studies [1,14,15]. The notable disparity here confirms that there is a significant ‘dark figure’ of violent victimisation, and suggests that people with serious mental illness may be less inclined to report even serious violent offences to the authorities.

### Relationship between victimisation, prior offending, and substance-use disorders

Confirming previous research, the current study demonstrated that the presence of a substance-use disorder significantly increases the risk of victimisation [15,16]. Indeed, patients with both a schizophrenia-spectrum and substance-use disorder were nearly four times more likely than community controls to have a record of violent crime victimisation, and over six times more likely to have a sexually violent victimisation record. They were also nearly twice as likely to have a victimisation record when compared to schizophrenia-spectrum patients without co-morbidsubstance-use disorders. However, substance misuse alone cannot explain this association, since the risk of victimisation was significantly higher among those with schizophrenia-spectrum disorders even when the presence of a co-morbidsubstance-use disorder was taken into account. Moreover, violent victimisation records were still significantly more common in schizophrenia-spectrum patients *without* co-morbid substance-use disorders than in controls.

A history of offending also increased the risk of victimisation in both the community and schizophrenia-spectrum groups. Community members who had offended were three times more likely than other community members to have a victimisation record, whilst the rates of official victimisation amongst offending schizophrenia-spectrum patients was more than 3.5 times greater than that of patients with no offence history. This finding supports previous research which shows that offending and victimisation are important reciprocal risk factors [17], and emphasises that the two categories are not mutually exclusive [34].

### Victimisation over time: the impact of deinstitutionalisation

One of the most alarming findings in this research is that official rates of victimisation in schizophrenia-spectrum patients appear to have risen dramatically over the period of deinstitutionalisation, an increase not paralleled in the general community. Indeed, whilst victimisation rates in the community have remained fairly constant over the past thirty years, the number of recorded victimisation incidents in the schizophrenia-spectrum sample has more than doubled.

There are three plausible explanations for this apparent increase. Firstly, one can hypothesise that the deinstitutionalisation process which occurred between the 1970s to mid -1990s has resulted in increasing numbers of mentally ill persons being exposed to risks within the community that they had previously been ‘sheltered’ from in institutional care, thus leading to a rise in reported victimisation rates. Indeed, a similar argument has been proposed to account for rising levels of homelessness [23,35]. In support of this theory, the completion of deinstitutionalisation in Australia in the mid-1990s [20] corresponds closely with a stabilisation in the rates of victimisation in schizophrenia-spectrum patients; this rate has now remained relatively constant for 15 years. A second related consideration relates to the decreases in the length of inpatient psychiatry admissions more recently and therefore the likelihood that the estimated rates of victimisation would have been an underestimate of the true prevalence that occurred in the inpatient environment prior to deinstitutionalisation. While police can be, and are, called upon when crimes are committed in inpatient settings, it is highly likely that a number of additional decision-making processes among hospital staff deterred action from being taken, due to their high thresholds for violent and otherwise antisocial behaviour that may normally lead to police involvement in community settings.

A third explanation for the increase focuses on changes in policing practice and social policy. Since the 1980s, there has been a greater focus on community well-being and engagement, and ‘community policing’ is now a well-established model in many countries. Through the implementation of community policing models, a number of initiatives have been developed to improve relationships with vulnerable populations. For example, in Victoria, all new police recruits are now trained in recognising the signs of mental illness and are given strategies for interacting and communicating with this population [36]. In some jurisdictions, mental health officers are employed to facilitate effective relationships between police and the mentally ill. Undoubtedly, this new approach has contributed to a more active ‘social welfare’ role for operational police [37], which may be reflected in an increasing number of schizophrenia-spectrum patients finding themselves in a position of reporting incidents of crime victimisation to the police.

## Conclusions

From the present research it is clear that people with schizophrenia-spectrum disorders have an increased vulnerability to violent crime victimisation, particularly if they have co-morbid substance problems or a history of offending. Taking the existing self-report literature into account, it appears that this population may experience higher than average rates of crime victimisation, but are less likely to report these experiences to the police. This makes the higher rates of recorded violent victimisation amongst schizophrenia-spectrum patients even more significant.

These results emphasise that when assessing a client’s history, clinicians should routinely enquire about victimisation experiences in schizophrenia-spectrum patients. Although it seems likely that police relations with the mentally ill have improved over time, similar recommendations could be made to police and security authorities, who may need to spend additional time and resources fostering positive relations with community members to encourage crime victimisation disclosure and recording of instances which are reported. Finally, victim service agencies may need to be tailored to those with severe mental illness who do not readily report crimes through official avenues, so that victims can access rehabilitative and supportive services, regardless of their psychiatric and offending histories.

Arguably the most startling finding presented here is that official victimisation rates amongst schizophrenia-spectrum patients in Victoria appear to have risen dramatically over the period of deinstitutionalisation. It has been argued elsewhere that the problems associated with deinstitutionalisation are not a result of the process *per se*, but rather of inadequate funding and support to sustain the model in the community over the long-term [38]. The present research provides some tentative support for this contention, and demonstrates the distinct possibility of an unintended but significant adverse outcome of deinstitutionalisation for people experiencing schizophrenia-spectrum disorders. Further research and a more detailed analysis of these trends (both in relation to violent and non-violent crime) are certainly warranted to further elucidate the nature and true extent of these victimisation experiences.

## Competing interests

The authors declare no competing interests. This study was supported by a linkage grant from the Australian Research Council (LP0774829); a collaboration between Monash University, the Victorian Institute of Forensic Mental Health and Victoria Police.

## Authors’ contributions

T.S. conducted the research as part of her doctoral thesis and wrote the manuscript. S.T., P.M., and J.O. were T.S.’s doctoral research supervisors, providing guidance and input throughout the research development stage. All three were involved in editing and revising the manuscript. S.L. acted as project manager and assisted with data coding. J.O. was listed as the chief investigator on the project grant. All authors read and approved the final manuscript.

## Pre-publication history

The pre-publication history for this paper can be accessed here:

http://www.biomedcentral.com/1471-244X/13/66/prepub
